# Real-World Evidence on the Effectiveness of Pembrolizumab in Patients With Recurrent/Metastatic/Unresectable Head and Neck Squamous Cell Cancer: A Systematic Review and Meta-Analysis

**DOI:** 10.7759/cureus.76709

**Published:** 2025-01-01

**Authors:** Meenakshi Meenu, Pravesh Dhiman, Muninder Kumar, Pranita Pradhan, Shivam Pandey

**Affiliations:** 1 Pharmacology, All India Institute of Medical Sciences, Bilaspur, Bilaspur, IND; 2 Medical Oncology, All India Institute of Medical Sciences, Bilaspur, Bilaspur, IND; 3 Radiation Oncology, All India Institute of Medical Sciences, Bilaspur, Bilaspur, IND; 4 Indian Council of Medical Research (ICMR) Advanced Center for Evidence Based Child Health, Postgraduate Institute of Medical Education and Research, Chandigarh, IND; 5 Biostatistics, All India Institute of Medical Sciences, New Delhi, New Delhi, IND

**Keywords:** head & neck cancer, immune check-point inhibitor, pembrolizumab, real-world evidence, systematic review

## Abstract

The aim of the review was to systematically review real-world data on the effectiveness and safety of pembrolizumab in recurrent/metastatic/unresectable head and neck squamous cell cancer (HNSCC) patients. Two independent reviewers retrieved the studies separately and simultaneously. PubMed, Embase, Scopus, Web of Science, and Cochrane Central were searched for prospective and retrospective studies on recurrent/metastatic/unresectable HNSCC patients treated with either pembrolizumab monotherapy or pembrolizumab combination therapy published till November 2024. Studies published in the English language and available as full-text articles were included while studies with patients treated with additional chemotherapeutic agents that do not include chemotherapy with fluorouracil, platinum, cetuximab, or radiotherapy were excluded. Outcomes were overall response rate, progression-free survival, overall survival, and adverse events. Forest plots were generated using RevMan software version 5.4 (2020; The Cochrane Collaboration, London, United Kingdom). The ROBINS-I (Risk Of Bias In Non-randomised Studies - of Interventions) tool was used for the assessment of the potential risk of bias and the quality of evidence was synthesized using GRADEpro (Evidence Prime Inc., Hamilton, Canada). We identified 5884 records, removed 2784 duplicate records, and screened 3100 studies. A total of 2931 records were excluded based on title/abstract. Of the remaining 169 articles, nine studies satisfied eligibility criteria and were included for final review. Pooled data suggested that 1006 patients were administered with pembrolizumab monotherapy while 448 patients received pembrolizumab combination therapy. Monotherapy improved the overall response rate compared to combination therapy. No significant difference in progression-free survival and overall survival was found between the two groups. Adverse events were reported less in pembrolizumab monotherapy compared to pembrolizumab combination therapy. The studies were assessed at high risk of bias and graded at low to very low quality of evidence. The study showed some beneficial effects of pembrolizumab monotherapy in recurrent/metastatic/unresectable HNSCC patients in real-world scenarios. However, more studies are required to generate robust evidence.

## Introduction and background

Head and neck squamous cell cancer (HNSCC) is one of the leading causes of cancer-related morbidity and mortality globally [1]. A large percentage of these patients progress to poor prognostic stage of recurrent or metastatic or unresectable cancer [2]. Before the advent of immune checkpoint inhibitors such as pembrolizumab, the standard of care for management included platinum-based therapy, cetuximab, 5-fluorouracil, or radiotherapy, which provided median overall survival of 10 months but with substantial toxicity [3-6]. Recent evidence strongly points towards a beneficial effect of pembrolizumab in the management of HNSCC [7]. Pembrolizumab is also used in combination with chemotherapy in HNSCC as chemotherapy alters the architecture of the cancer zone compromising host immune suppression by cancer cells leading to an increase in shedding of tumor antigen which curbs tumor growth. The substantial survival benefits of pembrolizumab, either alone or in combination with chemotherapy, compared to chemotherapy alone in HNSCC have brought a promising shift in the management of these patients [7,8].

In addition to survival benefits, pembrolizumab is mostly safer than chemotherapy except for activation of autoimmune-like reactions, including skin rashes, colitis, hepatitis, pneumonitis, and endocrine disorders such as thyroiditis [9,10]. In some cases, these adverse events may become life-threatening and demand urgent medical intervention. Such reactions can arise early during treatment or even after its completion. Managing immune-related adverse events often involves the use of immunosuppressive medications, such as corticosteroids, which may diminish the effectiveness and complicate the treatment process [9,10]. Another concern is the development of resistance. Patients who initially respond to pembrolizumab may develop resistance over time due to mutations in genes related to antigen presentation, loss of immune recognition, or adaptive resistance mechanisms by the tumor [11]. The affordability of these drugs is limited in low-resource settings.

Clinical trials assess new interventions in a controlled environment on select patient groups chosen using strict eligibility criteria, minimizing the impact of comorbidities and other confounding factors while routine clinical practice presents a myriad of conditions, which could alter the efficacy and safety profile of a drug [12-18]. The controlled settings of a trial promote better treatment adherence and thorough follow-up, especially for patients experiencing adverse or serious adverse events and levels of monitoring, while these conditions are harder to achieve in real-world practice. Nevertheless, real-world data analysis transforms the efficacy and safety results of a clinical trial into real-world effectiveness and risks, adapting the clinical trial data to a wider patient population [12-18]. There is a lack of comparative data and evident disparity between country-specific guidelines on the use of pembrolizumab monotherapy versus pembrolizumab combination chemotherapy in HNSCC patient cohort with PD-L1 CPS ≥1 in routine patient care [19,20]. Therefore, we conducted this systematic review and meta-analysis with the aim of analyzing real-world data on the effectiveness and safety profile of pembrolizumab in the management of HNSCC.

## Review

Review question

This review attempts to answer the question: What is the effectiveness and safety of pembrolizumab in patients with recurrent or metastatic or unresectable head and neck squamous cell cancer in real-world practice?

Methods

The PROSPERO (International Prospective Register of Systematic Reviews) guidelines [21] were followed and the protocol was registered on PROSPERO (registration number CRD42022373730). The Cochrane Handbook for Systematic Reviews of Interventions [22] and Preferred Reporting Items for Systematic Reviews and Meta-analyses (PRISMA) guidelines [23] were adhered to for the review. The studies eligible for review were cohort studies, prospective observational, retrospective observational, and case-control studies. Only full-text articles were included for systematic review and meta-analysis. Studies on recurrent or metastatic or unresectable HNSCC patients treated with either pembrolizumab monotherapy or pembrolizumab combination therapy (pembrolizumab plus chemotherapy) were eligible while studies with patients treated with additional chemotherapeutic agents that do not include chemotherapy with fluorouracil, platinum, cetuximab or radiotherapy were excluded. In addition, the studies with immune checkpoint inhibitors other than pembrolizumab and in which data exclusive to pembrolizumab could not be extracted were excluded. Cut-off levels for PD-L1 expression were not used as an inclusion criterion. Study groups included: (i) pembrolizumab combination therapy (pembrolizumab plus chemotherapy) and (ii) pembrolizumab monotherapy. Outcomes were overall response rate which is defined as the proportion of patients with partial responses plus complete responses according to the Response Evaluation Criteria in Solid Tumors (RECIST) criteria version 1.1, progression-free survival (PFS), overall survival, and adverse events. The definitions of outcomes were taken from the RECIST criteria version 1.1 [24].

Literature and Database Search

We conducted a systematic search on PubMed, Embase, Scopus, Web of Science, and Cochrane Central to extract data and information from the literature. We included studies published till November 2024. Only full-text articles published in the English language were included for review. The following keywords were used: Head and Neck Cancer, Head and Neck Squamous Cell Cancer, HNSCC, Metastatic Head and Neck Cancer, Recurrent Head and Neck Cancer, Unresectable Head and Neck Cancer, Pembrolizumab, Programmed Death Receptor-1 Blocking Antibody, PDL1 Blocking Antibody, Programmed Death Receptor-1 Inhibitor, Immune Check Point Inhibitor. Following search terms were used - (((((("Head and neck cancer" OR "Head and neck squamous cell cancer" OR " HNSCC" OR "recurrent head and neck cancer" OR"unresectable head and neck cancer" OR "metastatic head and neck cancer")) OR ("Head and Neck Neoplasms"[Mesh])) OR ("head neck neoplasm")) OR ("neck neoplasm")) OR ("head neoplasm")) AND (((Pembrolizumab OR "programmed death receptor-1 blocking antibody" OR "PDL1 blocking antibody" OR "programmed death receptor-1 inhibitor" OR "PDL1 inhibitor" OR "immune check-point inhibitor")) OR ("pembrolizumab" [Supplementary Concept])).

Data Extraction

Two independent reviewers retrieved the studies along with cross-referencing and hand searching separately and simultaneously with the help of the information specialist. The studies were filtered according to eligibility criteria and duplicate studies were removed. No conflict was identified among the reviewers. The following information was extracted for each study: author, year of publication, study design, study site, study duration, sample size in each group, inclusion criteria, exclusion criteria, study groups, overall response rate, PFS, overall survival, adverse event, follow-up duration and any other relevant additional information (Appendix A).

Data Analysis

For dichotomous variables, data were pooled and represented as the number of events out of the total number of participants in each group. The odds ratio (OR) with the corresponding 95% confidence interval (CI) and a p-value <0.05 were used to compare groups with respect to various clinical outcomes and statistical significance. Heterogeneity was evaluated using the I2 statistic. Forest plots were visually inspected to determine the overlap of confidence intervals. I^2^ value and small-study effect were taken into consideration to determine the type of model i.e., fixed or random effect model for data analysis. For I^2^>50%, the random effect model and I^2^<50%, the fixed effect model with the Mantel-Haenszel method was to be used along with careful evaluation of small-study effects. In addition, sensitivity analysis was performed for high heterogeneity or inconsistency. To explore possible publication biases, a funnel plot was derived, and the plot’s symmetry was visually assessed. The stability of the results was obtained by sensitivity analyses with sequential elimination of results of each study. All analyses were done using RevMan version 5.4 (2020; The Cochrane Collaboration, London, United Kingdom).

Sensitivity Analysis

To check for the robustness of results, the fixed and random effect models were interchanged for individual outcome parameters, and effect sizes were compared in sensitivity analyses. OR was replaced with risk ratio (RR) and then risk difference and change in the direction of the effect estimate were noted. Subsequently, for sensitivity analysis of the effect of high or unclear risk of bias in any domain in a study, such studies were individually removed and then any change in the direction of the summary estimate of effect was noted.

Assessment of Risk of Bias

Two authors independently assessed the quality of studies and the potential risk of bias using the ROBINS-I tool for risk of bias assessment [25]. ROBINS-I tool is applicable for observational designs such as cohort and case-control studies, where intervention groups are determined during routine treatment decisions mimicking real-world population contexts. When compared to other tools such as the Newcastle-Ottawa Scale, the ROBINS-I (Risk Of Bias In Non-randomised Studies - of Interventions) tool comes with detailed manuals to allow consistent interpretation of results by different users.

The following ROBINS-I criteria were used: (i) Selection of participants, (ii) Confounding variables, (iii) Intervention (exposure) measurement, (iv) Blinding of outcome assessment, (v) Incomplete outcome data, and (vi) Selective outcome reporting. The evaluations within each domain contribute to an overall risk of bias determination across all domains for the specific outcome under assessment [25].

Quality of Evidence Synthesis

GRADEpro tool (Evidence Prime Inc., Hamilton, Canada) was applied for quality of evidence synthesis for all domains of GRADE criteria for assessing the certainty in the evidence (i.e., risk of bias, imprecision, inconsistency, indirectness, publication bias, large effects, dose-response gradients, and residual plausible opposing bias) [26].

Results

Screening of Studies and Data Extraction

**Figure 1:**
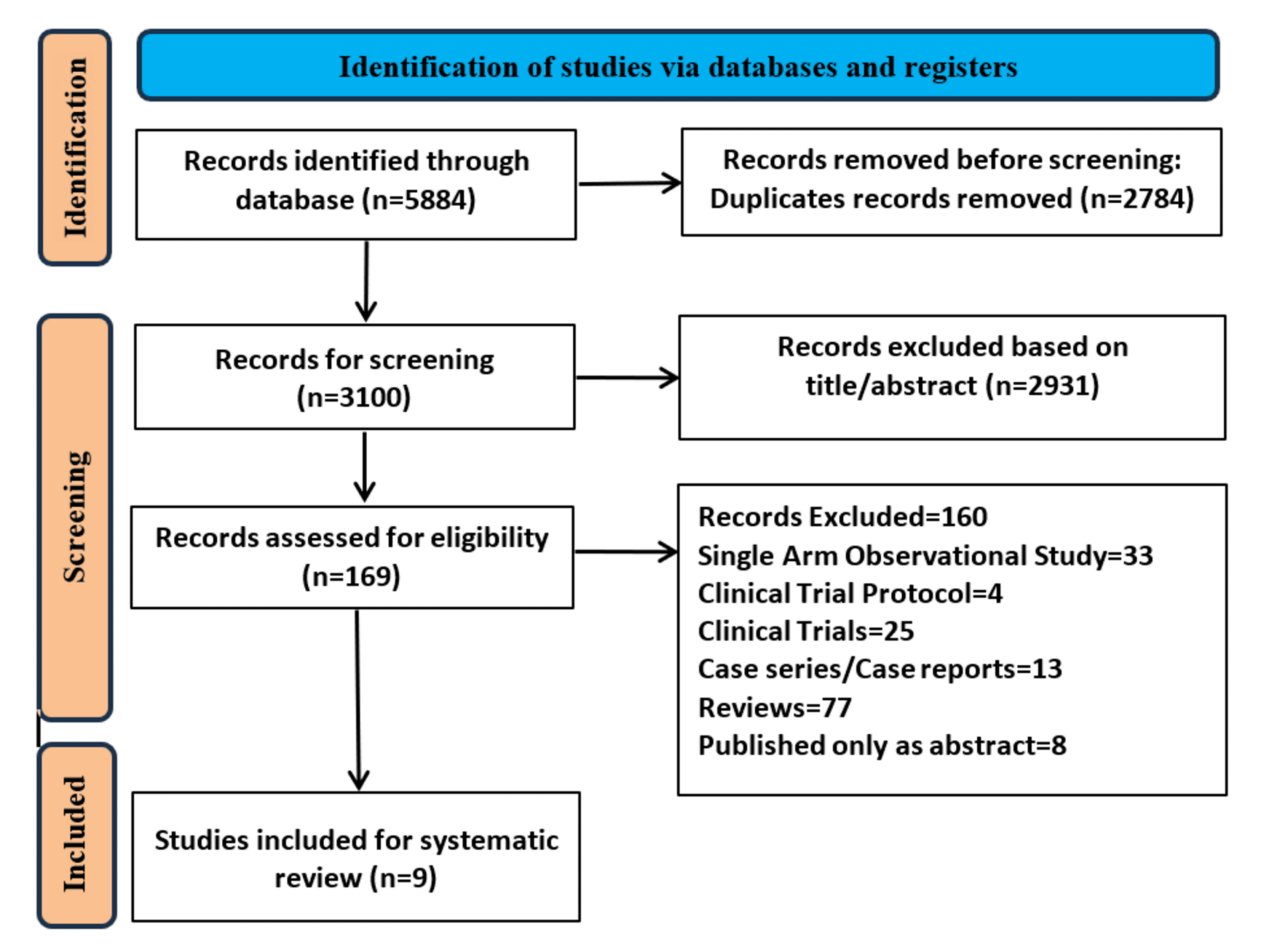
PRISMA flowchart showing the study selection process PRISMA: Preferred Reporting Items for Systematic Reviews and Meta-Analysis

A detailed search of PubMed, Embase, Scopus, Web of Science, and Cochrane Central identified 5884 records (Appendix B). We removed 2784 duplicate records and screened 3100 records. A total of 2931 records were excluded after reviewing the title and the abstract of the studies. Out of the remaining 169 studies, 77 were reviews, 33 studies did not have any control group, 13 studies were case series/case reports, four studies were clinical trial protocols, and 25 studies were clinical trials. Eight studies were only published as abstracts and hence excluded from the review [27-34]. Nine studies satisfied our eligibility criteria and were included in the review [35-43]. The detailed search results are illustrated in the PRISMA flow chart (Figure 1). Characteristics of the included studies have been detailed in Table 1. There were 1006 patients in the pembrolizumab group while 448 patients were in the pembrolizumab plus chemotherapy group.

**Table 1:** Characteristics of the studies included for review and meta-analysis. AE: adverse events; CI: confidence interval; DCR: disease control rate; ECOG PS: Eastern Cooperative Oncology Group performance status; HNSCC: head and neck squamous cell cancer; HPV: human papilloma virus; HR: hazard ratio; ICI: immune checkpoint inhibitor; IRAE: immune-related adverse events; IQR: interquartile range; ORR: overall response rate; OS: overall survival; P: pembrolizumab; C: chemotherapy; PD-L1: programmed death ligand-1; PFS: progression-free survival; R/M/U: recurrent/metastatic/unresectable

Author, Year	Country	Duration of study	Design of study	Inclusion criteria	Exclusion Criteria	Intervention Pembrolizumab (P)	Control Pembrolizumab + Chemotherapy (P+C)	Outcome	Additional information
Black et al., 2023 [35]	United States	2019- 2021	Real-world retrospective cohort study	1. Age ≥ 18 years; 2. Histologically or cytologically confirmed R/M/U HNSCC	1. Prior platinum therapy during 6 months of first-line Pem therapy; 2. Primary cancers before diagnosis of R/M/U HNSCC; 3. Received treatment with a new investigational agent.	n=431	n=215	OS	HPV-+ tumor status and lower ECOG PS were associated with longer, and oral cavity tumor site with shorter real-world OS
Cirillo et al 2024 [36]	Italy	2021-2023	Real-world retrospective cohort study	R/M/U HNSCC CPS ≥1	Not mentioned	n=57	n=35; Younger patients, patients had lower ECOG PS scores and had less previous exposure to radio-chemotherapy.	PFS, OS	Median follow up for 12 months.
Hobday et al., 2022 [37]	United States	2015-2021	Real-world retrospective cohort study	1. Age ≥ 18 years; 2. Treated with ICI-based therapy for R/M HNSCC	1. Received immunotherapy for melanoma, cutaneous squamous cell carcinoma, and basal cell carcinoma of the head and neck; 2. Treated with Pembrolizumab as part of a clinical trial	n=126	n=23	OS and PFS	Median OS=12.9 months, median PFS=3.9 months. ECOG performance status greater than 1 (HR, 2.720; 95% CI, 1.866-3.964) were associated with worse OS.
Iwaki et al., 2023 [38]	Japan	2020-2022	Real world retrospective cohort study	1. Pathologically diagnosed as squamous cell carcinoma; 2. primary sites included oral cavity, oropharynx, hypopharynx, nasal/paranasal cavity, and larynx; 3. recurrent or metastatic, unresectable disease; and 4. first line treatment using P or P+C.	Not mentioned	n=31	n=23	OS and PFS	Median follow-up duration was 11.6 months.
Matsuo et al., 2023 [39]	Japan	2020- 2022	Real world retrospective cohort study	1. R/M-HNSCC; 2. Histological confirmation of squamous cell carcinoma; 3. Had received either P or P+C.	Not mentioned	n=87	n=52	PFS and OS.	Median duration of observation was 10.8 months.
Nakano et al., 2022 [40]	Japan	2022	Real world retrospective cohort study	1. Age ≥ 18 years; 2. Histologically or cytologically confirmed R/M/U HNSCC.	Received treatment with a clinical trial drug.	n=60	n=37	1-year OS, ORR and serious (≥Grade 3) AEs	
Okada et al., 2023 [41]	Japan	2019-2022	Real-world retrospective cohort study	1. Patients with squamous cell carcinoma of head and neck including nasopharynx, paranasal sinuses, and salivary glands; 2. Patients treated with either pembrolizumab or pembrolizumab with chemotherapy.	1. Patients with CPS<1 who were more likely to receive pembrolizumab-free regimens; 2. Patients with non-squamous cell carcinoma 3. Patients included in clinical trial	n=124	n=30	OS, PFS, ORR, DCR and IRAE	DCR in monotherapy – 72 (58.1%); Combination therapy – 22 (73.3%).
Sano et al., 2022 [42]	Japan	January 2020 and January 2022	Real-world retrospective cohort study	1. 20 years or older; 2. Histologically confirmed R/M HNSCC; 3. Treated with pembrolizumab as first-line therapy; 4. Treatment response was recorded by CT scans at least once; 5. ECOG PS of 0-2.	Previously received immunotherapy or chemotherapeutic regimen as first-line therapy	n=14	n=18	1-year OS, PFS, ORR, IRAE.	Median follow up duration was 9.8 months. Kaplan–Meier analysis showed that patients with favourable objective responses and an Eastern Cooperative Oncology Group performance status of 0 had longer survival.
Thapa et al., 2024 [43]	Scotland	March 2020 and 30 September 2021	Real-world retrospective cohort study	Patients treated with either pembrolizumab or pembrolizumab with chemotherapy.	Patients who received pembrolizumab as part of a clinical study	n=76	n=15	OS, PFS, Duration of response, IRAE.	Median follow-up was 10.8 months.

Overall Response Rate

**Figure 2:**
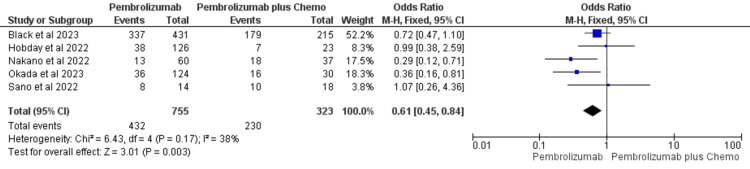
Forest Plot for overall response rate comparing pembrolizumab with pembrolizumab plus chemotherapy in recurrent/metastatic/unresectable HNSCC References: Black et al., 2022 [35], Hobday et al., 2022 [37], Nakano et al., 2022 [40], Okada et al. [41], and Sano et al., 2022 [42] HNSCC: head and neck squamous cell cancer

The overall response rate was reported by five studies included in the review [35,37,40-42]. A total of 432 events were recorded out of 755 patients in the pembrolizumab group while 230 events were recorded out of 323 patients in the pembrolizumab plus chemotherapy group. Heterogeneity I^2^ was 38% and the influence of small-study effects was not seen in the data analysis. Moreover, there was only one large study with 52.2% weight [35], and it also did not influence the results in any one direction. Therefore, the fixed effect model was used for pooled data analysis. OR was in favor of pembrolizumab suggesting an improved overall response rate with pembrolizumab (OR = 0.61, 95%CI: 0.45, 0.84) (Figure 2).

PFS

**Figure 3:**
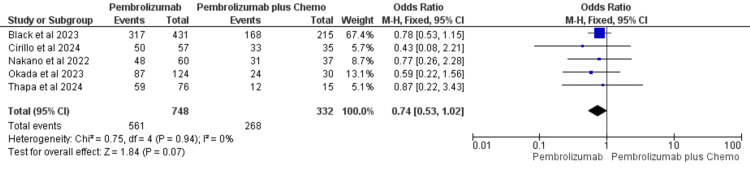
Forest plot for progression-free survival comparing pembrolizumab with pembrolizumab plus chemotherapy in recurrent/metastatic/unresectable HNSCC References: Black et al., 2023 [35], Cirillo et al., 2024 [36], Nakano et al., 2022 [40], Okada et al., 2023 [41], and Thapa et al., 2024 [43] HNSCC: head and neck squamous cell cancer

Five studies reported PFS. In the pembrolizumab monotherapy group [35,36,40,41,43]; 748 patients were included, with 561 events recorded, while the pembrolizumab plus chemotherapy group had 332 patients and 268 events. The pooled data showed no heterogeneity (I²=0%) or evidence of small-study effects. Excluding a study with a weight of 67.4% [35] did not alter the results. Consequently, a fixed-effect model was applied for analysis. Although the OR favored pembrolizumab monotherapy over pembrolizumab plus chemotherapy, the confidence interval crossed 1 (OR=0.74, 95% CI: 0.53, 1.02), indicating that the finding was not statistically significant (Figure 3).

Overall Survival

**Figure 4:**
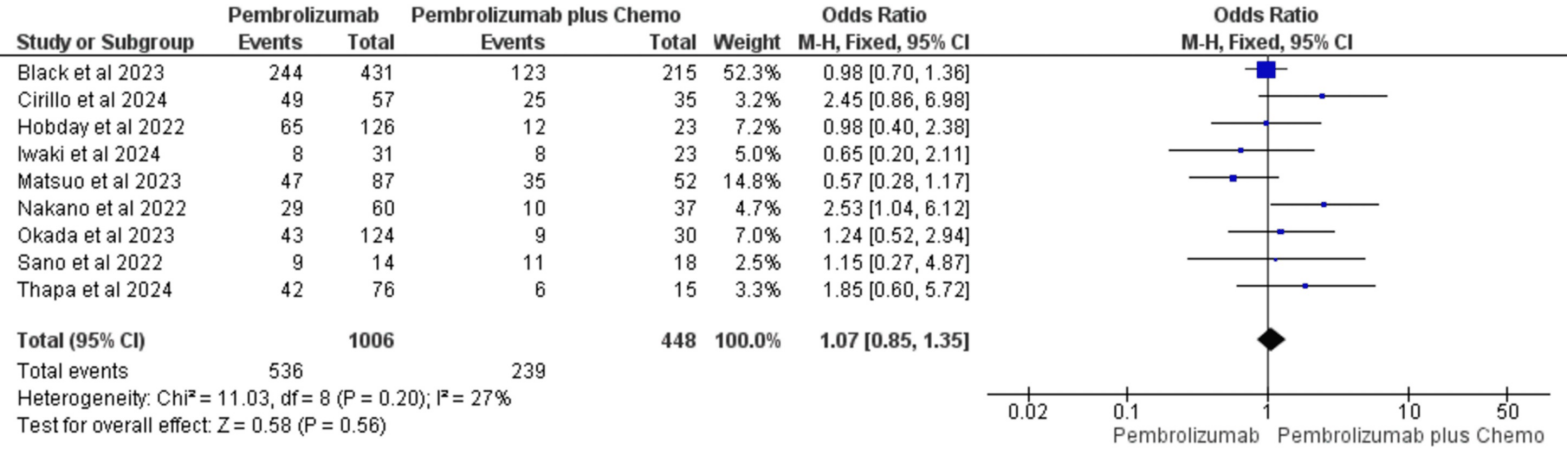
Forest plot for overall survival comparing pembrolizumab with pembrolizumab plus chemotherapy in recurrent/metastatic/unresectable HNSCC References: Black et al., 2022 [35], Cirillo et al., 2024 [36], Hobday et al., 2022 [37], Iwaki et al., 2024 [38], Matsuo et al., 2023 [39], Nakano et al., 2022 [40], Okada et al., 2023 [41], Sano et al., 2022 [42], and Thapa et al., 2024 [43] HNSCC: head and neck squamous cell cancer

Overall survival was recorded by all studies included in the review [35-43]. In the pembrolizumab monotherapy group, 1006 patients were included, with 536 events recorded, while the pembrolizumab plus chemotherapy group had 448 patients and 239 events. Though heterogeneity was moderate (I^2^=27%), the influence of small-study effects was not seen in the data. In addition, the study with maximum weight (52.3%) did not pull the results in either direction. Therefore, a fixed effect model was used. Pooled data indicated that overall survival was equivocal with either pembrolizumab monotherapy or combination therapy (OR=1.07, 95%CI: 0.85, 1.35) (Figure 4).

Adverse Events

**Figure 5:**
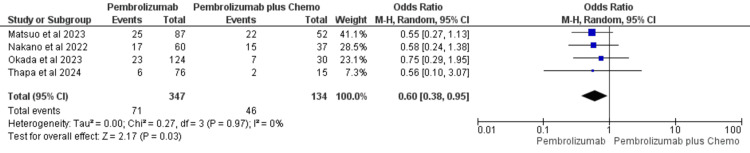
Forest plot for adverse event comparing pembrolizumab with pembrolizumab plus chemotherapy in recurrent/metastatic/unresectable HNSCC References: Matsuo et al., 2023 [39], Nakano et al., [40], Okada et al., 2023 [41], and Thapa et al., 2024 [43] HNSCC: head and neck squamous cell cancer

Four studies reported adverse events with respect to each group [39-41,43]. Seventy-one adverse events were reported in pembrolizumab monotherapy (n=347) as compared to 46 adverse events in pembrolizumab plus chemotherapy group (n=134). The pooled data showed no heterogeneity (I²=0%) or evidence of small-study effects. Therefore, a fixed-effect model was applied for the analysis of pooled data. OR was in favor of pembrolizumab monotherapy compared to the pembrolizumab plus chemotherapy group (OR=0.60, 95%CI: 0.38-0.95) (Figure 5).

Cirillo et al. documented immune-related toxicities in 18.5% (n=17) of patients but did not provide data for specific groups [36]. Similarly, Hobday et al. reported treatment-related adverse events in 50 out of 212 patients without detailing adverse events for individual groups [37]. Thyroid function abnormalities occurred in 22 patients, rash in 15, and colitis in nine. Sano et al. found that 50% (n=16) of patients experienced immune-related adverse events, with four patients having grade 3, six having grade 2, and another six having grade 1 adverse events [42]. Black et al. [35] and Iwaki et al. [38] did not report adverse event data. Table 2 gives a concise representation of adverse event data.

**Table 2:** Adverse event recorded in the studies included in the systematic review. * Only immune-related toxicities were reported and data for individual groups was not mentioned; **Data for individual groups was not mentioned

Study	Adverse events, n (%)	
Cirillo et al., 2024 [36]	17 (18.5)*	
Hobday et al., 2022 [37]	50 (27.3)**	
Sano et al., 2022 [42]	16 (50), Grade ≥3 = 4 (12.5)	
Black et al., 2023 [35]	Not reported	
Iwaki et al., 2024 [38]	Not reported	

Publication Bias

**Figure 6:**
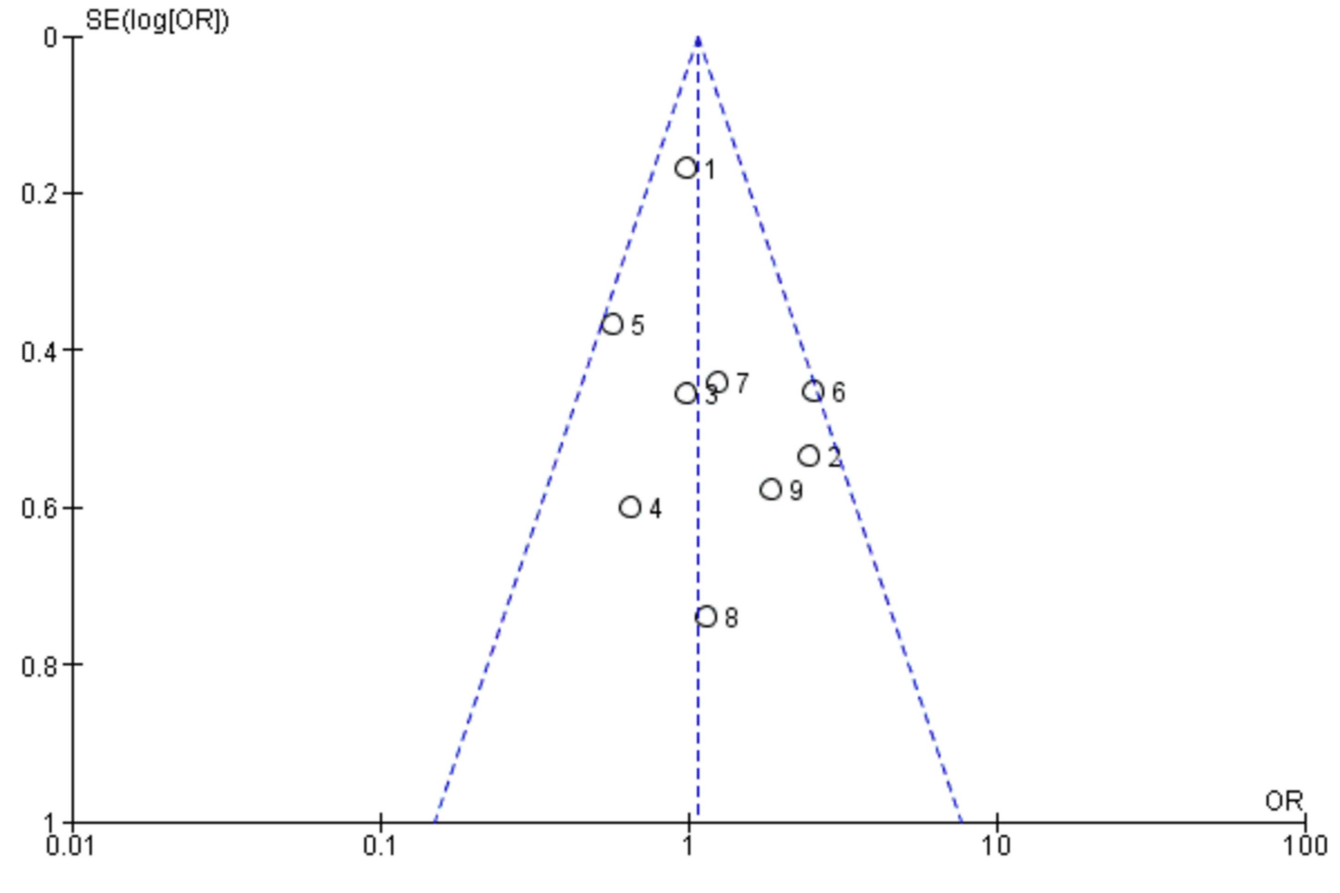
Funnel plot for assessing publication bias in studies included in the review. Figure was generated using RevMan version 5.4 (2020; The Cochrane Collaboration, London, United Kingdom) References: [35-43] OR: odds ratio; SE: standard error

No publication bias was detected on a visual inspection of the funnel plot which showed a symmetric distribution of studies (Figure 6).

Risk of Bias Assessment Using the ROBINS-I Tool

**Figure 7:**
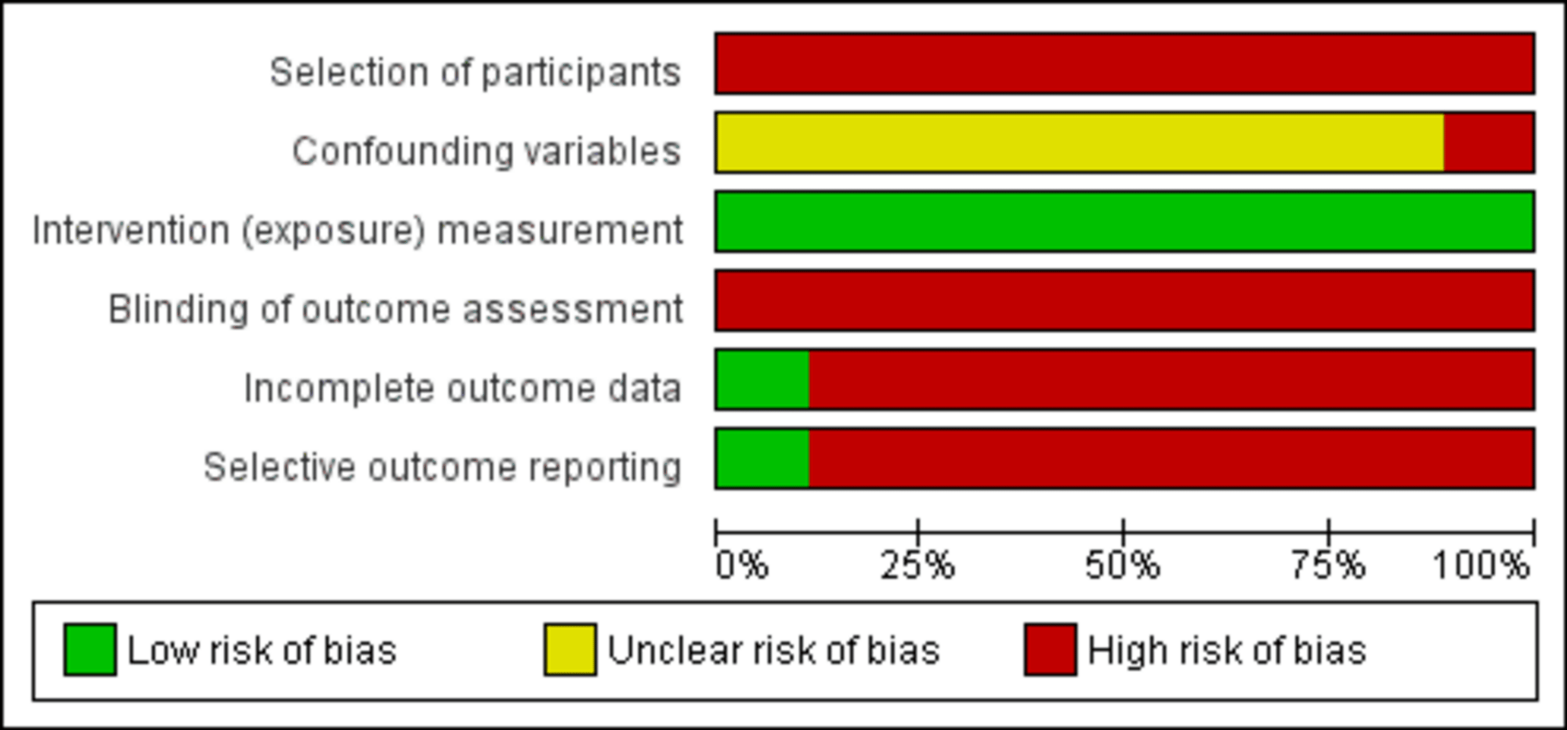
Risk of bias graph for studies included in the review. Figure was generated using RevMan version 5.4 (2020; The Cochrane Collaboration, London, United Kingdom) References: [35-43]

**Figure 8:**
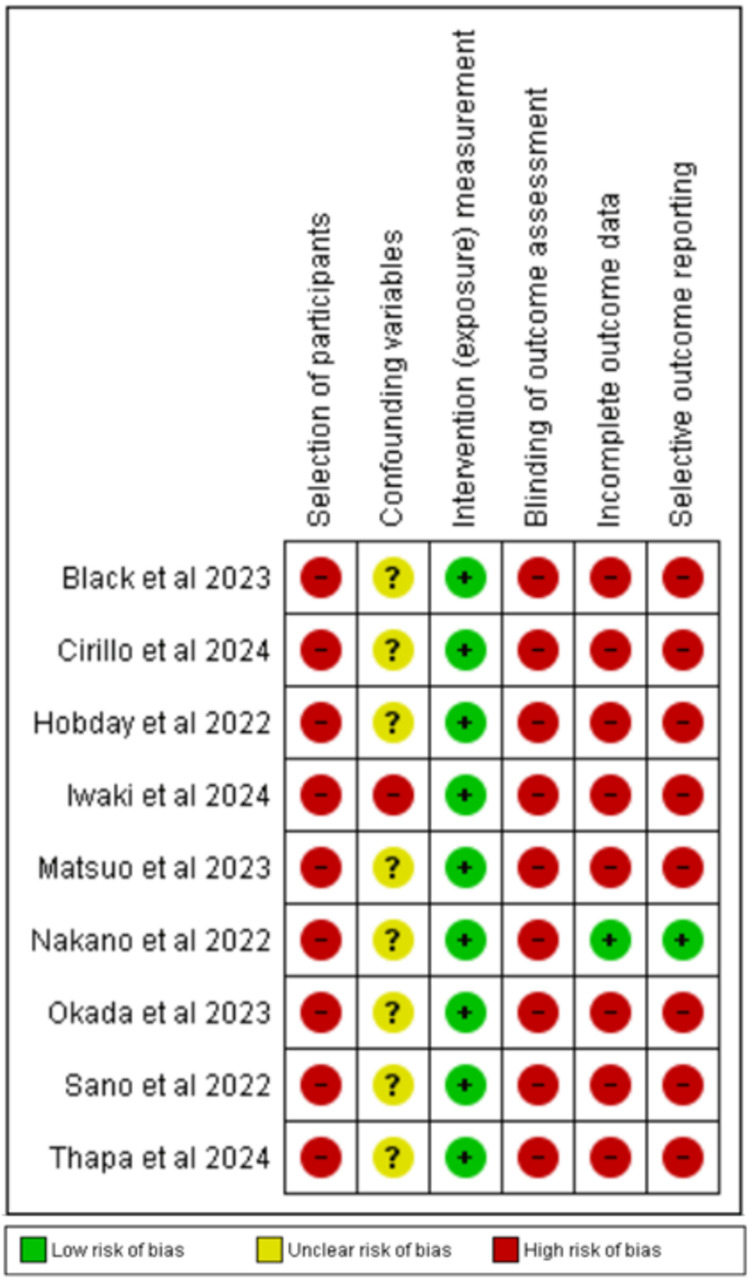
Risk of bias summary for studies included in the review. Figure was generated using RevMan version 5.4 (2020; The Cochrane Collaboration, London, United Kingdom) References: [35-43]

As all studies were non-randomized, these were assessed at high risk for selection bias. Blinding was not done in any of the studies and hence assessed at high risk of bias for outcome assessment. Though studies have identified factors that were associated with poor outcomes and baseline characteristics of studies were reported and analyzed, baseline matching was not done, resulting in an unclear risk of bias for confounding variables except for one study [38], which was assessed as high risk of bias. A study with all outcomes reported was considered to have low risk while studies wherein all outcomes were not reported or incompletely reported, were assessed at high risk for incomplete outcome data and selective outcome reporting biases. As overall response rate, PFS, and overall survival are hard endpoints, thus intervention (exposure) measurement was assessed at low risk of bias. No discrepancy was identified among the reviewers. Domain, description, and risk of bias were analyzed using the ROBINS-I tool. Risk of bias is summarised in Figure 7 and a graphical presentation is shown in Figure 8.

Quality of Evidence Using the GRADEpro Tool

The pooled studies were evaluated for the quality of evidence following the guidelines outlined in the Cochrane Handbook for Systematic Reviews of Interventions. Due to the retrospective nature of all included studies, the risk of bias was deemed ‘very serious,’ resulting in a downgrading of the quality of evidence by two levels across all outcomes. An additional one-level downgrade was applied for imprecision, while no indirectness was identified in the outcome measures. Based on the I² statistic and visual assessment of confidence interval overlap in the forest plot, heterogeneity was categorized as low for 'PFS' and 'adverse event' outcomes, and moderate for 'overall survival' and 'overall response rate.' The overall quality of evidence was rated as ‘very low’ for 'overall survival' and 'overall response rate’ outcomes and ‘low’ for 'PFS' and 'adverse event' outcomes using the data synthesized from the included studies. The assessment was performed using the GRADEpro tool, addressing all domains of the GRADE criteria (Table 3).

**Table 3:** Quality of evidence synthesis using GRADEpro tool* for evidence synthesis ^a ^The studies are observational cohort design and suffers from sampling, selection, allocation, recall, reporting and other biases as per ROBINS-I tool for risk of bias assessment; ^b^ Moderate heterogeneity;^ c^ Studies are not symmetrically distributed around either of the point estimate. The confidence intervals of studies crossed midline. Confidence intervals for estimates of test accuracy are wide which lowered the quality of evidence. *2020; The Cochrane Collaboration, London, United Kingdom CI: confidence interval; OR: odds ratio

Certainty assessment							Number of patients		Effect		Certainty
Number of studies	Study design	Risk of bias	Inconsistency	Indirectness	Imprecision	Other considerations	Pembrolizumab plus Chemotherapy	Pembrolizumab monotherapy	Relative (95% CI)	Absolute (95% CI)	
Overall Response Rate											
5	non-randomised studies	very serious^a^	serious^b^	not serious	serious^c^	all plausible residual confounding would reduce the demonstrated effect	432/755 (57.2%)	230/323 (71.2%)	OR 0.61 (0.45 to 0.84)	111 fewer per 1,000 (from 185 fewer to 37 fewer)	⨁◯◯◯ Very low^a,b,c^
Progression Free Survival											
4	non-randomised studies	very serious^a^	not serious	not serious	serious^c^	all plausible residual confounding would reduce the demonstrated effect	561/748 (75.0%)	268/332 (80.7%)	OR 0.74 (0.53 to 1.02)	51 fewer per 1,000 (from 118 fewer to 3 more)	⨁⨁◯◯ Low^a,c^
Overall Survival											
9	non-randomised studies	very serious^a^	serious^b^	not serious	serious^c^	all plausible residual confounding would reduce the demonstrated effect	536/1006 (53.3%)	239/448 (53.3%)	OR 1.07 (0.85 to 1.35)	17 more per 1,000 (from 41 fewer to 73 more)	⨁◯◯◯ Very low^a,b,c^
Adverse Events											
3	non-randomised studies	very serious^a^	not serious	not serious	serious^c^	all plausible residual confounding would reduce the demonstrated effect	71/347 (20.5%)	46/134 (34.3%)	OR 0.60 (0.38 to 0.95)	105 fewer per 1,000 (from 178 fewer to 11 fewer)	⨁⨁◯◯ Low^a,c^

Sensitivity Analysis

Upon replacing the fixed-effect to random-effect model and summary effect measure (OR or RR), no alteration in the direction of the summary estimate of effect was observed. 

Combined Positive Score (CPS) score and PFS

Few studies reported PFS in patients of different CPS scores grouped as (i) CPS score <1, (ii) CPS score 1-19, and (iii) CPS score ≥20. The comparative analysis showed that a higher percentage of patients with a CPS score >1 reached 1-year PFS as compared to patients with a CPS score <1 and the percentage was even higher in patients with a CPS score ≥20 compared to patients with a CPS score 1-19 (Table 4).

**Table 4:** Comparison of one-year progression-free survival rate of patients in groups with CPS <1, 1-19, and ≥20. CPS: combined positive score

Author, Year	CPS 1	CPS 1-19	CPS score ≥20
Hobday et al., 2022 [37]	40 (16%)	39 (37%)	34 (40%)
Matsuo et al., 2024 [39]	39.5 (13%)	61.6 (58%)	61.5 (60%)
Nakano et al., 2022 [40]	0 (3%)	60.7 (28%)	61.8 (97%)

In the study conducted by Black et al., patients with higher CPS scores were more likely to receive pembrolizumab monotherapy than pembrolizumab combination therapy [35]. Cirillo et al. reported improved PFS in the CPS ≥20 group compared to the CPS 1-19 group (log-rank p = 0.005, hazard ratio (HR): 0.50; 95%CI: 0.31-0.82) and better overall survival in both groups (log-rank p = 0.04, HR: 0.57; 95%CI: 0.33-0.98) [36]. Similarly, Okada et al. found that the median PFS was 7.3 months (95%CI: 3.1-12.0) and 5.8 months (95%CI: 1.6-7.6) for CPS 1-20 and CPS ≥20 groups respectively with no statistical significance (HR: 1.38; 95%CI: 0.52-3.67; p=0.52) [41]. Thapa et al. reported HR of 1.45 (CI: 0.67-3.02) for overall survival and 1.73 (CI: 0.87-3.36) for PFS in patients with CPS≥20 upon multivariable analysis [43].

Effect of Comorbidities and Other Clinical Variables

Black et al. found that patients with human papillomavirus (HPV)-positive tumors and lower Eastern Cooperative Oncology Group performance status (ECOG PS) had longer overall survival, while those with oral cavity tumors had shorter survival in the monotherapy group [35]. In the combination therapy group, HPV-positive tumor status was also linked to improved overall survival. Cirillo et al. reported that higher ECOG PS scores were associated with worse PFS (log-rank p = 0.004) and overall survival (log-rank p = 6e-04) [36]. Hobday et al. observed that patients with oral cavity tumors experienced poorer overall survival (HR: 0.567; 95%CI: 0.335-0.960) compared to those with tumors at other sites (HR: 0.491; 95%CI: 0.298-0.810) [37]. Additionally, a T4 tumor category at presentation (HR: 1.594; 95%CI: 1.062-2.394) and ECOG PS >1 (HR: 2.720; 95% CI: 1.866-3.964) were associated with worse survival outcomes. Similarly, Matsuo et al. reported that patients with an ECOG PS of 2-4 had significantly poorer overall survival compared to those with ECOG PS 0-1 (HR: 3.683; 95%CI: 2.083-6.513) [39]. Nakano et al. noted significantly better survival outcomes in patients with ECOG PS 0-1 compared to those with ECOG PS 2 in the pembrolizumab monotherapy group (p = 0.0037 and p = 0.0004, respectively), though these results were not significant in the pembrolizumab combination therapy group (p = 0.09) [40]. Sano et al. also reported that patients with ECOG PS 1 or 2 had worse overall survival compared to those with ECOG PS 0 [42]. Furthermore, survival was significantly longer in patients with ECOG PS 0 (HR: 0.21; 95%CI: 0.05-0.87; p = 0.032). In the study done by Thapa et al., the overall survival was poorer in ECOG PS 2 (HR: 3.95, 95%CI:1.06,13.47) than in ECOG PS 0 (HR: 0.46, 95%CI: 0.17, 1.10) [43].

Discussion

We found a limited number of clinical trials and retrospective studies on pembrolizumab in head and neck cancer patients. In one of the major clinical trials, KEYNOTE-040, 247 patients were assigned to the pembrolizumab group, while 248 received standard-of-care treatment [7]. The median overall survival in the intention-to-treat population was 8.4 months (95% CI: 6.4-9.4) for pembrolizumab and 6.9 months (95% CI: 5.9-8.0) for the standard of care (HR: 0.80, 95%CI: 0.65-0.98; nominal p=0.0161). The number of patients treated with pembrolizumab experienced lesser grade ≥3 treatment-related adverse events compared to those receiving standard care (13% vs. 36%). Another landmark clinical trial was a phase 3 study, KEYNOTE-048, which evaluated pembrolizumab alone and in combination with chemotherapy with an EXTREME drug regime of platinum-based therapy, cetuximab, and 5-fluorouracil for recurrent /metastatic /unresectable HNSCC [44,45]. Patients were randomly allocated in a 1:1:1 ratio to receive pembrolizumab, pembrolizumab - chemotherapy, or cetuximab - chemotherapy and efficacy was assessed across three populations i.e., those with a CPS ≥20, CPS ≥1, and the overall population. With a four-year follow-up, first-line pembrolizumab and pembrolizumab-chemotherapy continued to demonstrate a survival advantage over cetuximab chemotherapy in recurrent/metastatic HNSCC.

To supplement the evidence generated in clinical trials with real-world results, we conducted this review wherein nine real-world studies were included with 1006 and 448 patients in pembrolizumab monotherapy and pembrolizumab combination therapy groups respectively [35-43]. Based on the available evidence, pembrolizumab was found to improve the overall response rate in HNSCC patients with OR favoring monotherapy over combination therapy. For PFS, the CI (OR: 0.74; 95%CI: 0.53, 1.02) touched the central line, indicating comparable survival in both groups. Clinical trials had reported that PFS was enhanced with pembrolizumab in the PD-L1 CPS ≥20 (HR: 0.64; 95%CI: 0.48-0.84) and CPS ≥1 (HR: 0.79; 95%CI: 0.66-0.95) populations, and with pembrolizumab plus chemotherapy in the PD-L1 CPS ≥20 (HR: 0.64; 95%CI: 0.48-0.86), CPS ≥1 (HR: 0.66; 95%CI: 0.55-0.81). In the KEYNOTE-048 Japanese subgroup analysis, Takahashi et al. reported similar median PFS for monotherapy and combination therapy [46].

In our pooled data, overall survival was equivocal in monotherapy (n=1006) and combination therapy (n=448) groups with OR of 1.07 (CI: 0.85, 1.35). Our finding aligned with KEYNOTE-048 results, which also reported significant improvement in overall survival with monotherapy in the programmed death ligand 1 (PD-L1) CPS ≥20 group (HR: 0.61; 95%CI: 0.46-0.81) and CPS ≥1 group (HR: 0.74; 95%CI: 0.61-0.89) [44,45]. Pembrolizumab combination therapy also showed improved overall survival in the PD-L1 CPS ≥20 (HR: 0.62; 95%CI: 0.46-0.84), CPS ≥1 (HR: 0.64; 95% CI: 0.53-0.78), suggesting equivocal response of both groups in KEYNOTE-048 trial.

In the current review, 71 adverse events were reported in pembrolizumab monotherapy (n=347) and 46 events in pembrolizumab plus chemotherapy (n=134) with OR significantly supporting the monotherapy group (0.60; 95%CI: 0.38, 0.95). Our finding is similar to other studies such as Takahashi et al., who found a higher percentage of grades 3/5 treatment-related adverse events with pembrolizumab combination therapy than with pembrolizumab monotherapy [43]. In their Asian subgroup analysis, pembrolizumab monotherapy was associated with fewer adverse events than combination therapy (22% vs. 76% respectively) [43]. Immune-related adverse events most likely are mechanistically associated with immune checkpoint inhibitors such as pembrolizumab and can be seen in up to 70% of patients treated with immune checkpoint inhibitors [47,48].

Okada et al. [41] and Thapa et al. [43] reported that survival was better in patients with immune-related adverse events than in patients without immune-related adverse events.

Most studies reported longer survival with HPV-positive tumors and lower ECOG PS status. A higher percentage of patients with a CPS score >1 reached 1-year PFS as compared to patients with a CPS score <1 and the percentage was even higher in patients with a CPS score ≥20 compared to patients with a CPS score of 1-19.

To sum it up, the real-world evidence in our study supported the post hoc analysis of efficacy in terms of PFS and overall survival outcomes in KEYNOTE-040 and 048 clinical trials. Though clinical trials showed a clear survival benefit with pembrolizumab over chemotherapy, we did not find any clinical trial that reported a head-to-head comparison of pembrolizumab monotherapy with pembrolizumab, combination therapy. On the other hand, real-world studies accounted for a direct comparative analysis of pembrolizumab monotherapy with pembrolizumab combination therapy for overall response and patients’ survival.

In addition to inbuilt biases due to the retrospective design of the studies included for analysis, clinical heterogeneity on a range of factors such as matching of age and sex, comorbidities, tumor burden, HPV status, ECOG PS, and PDL1 CPS could have influenced the results of this review. An additional source of bias in the studies reviewed arose from the treatment pattern, where patients with CPS ≥20 were predominantly given monotherapy, while those with CPS 1-19 were treated with combination therapy. This approach was driven by clinicians' preference to administer cytotoxic anticancer drugs alongside treatment in patients with low CPS. Preferential use of treatment led to selection bias in the analysis. Real-world studies often struggle with maintaining internal validity due to biases inherent in their design and implementation. The absence of randomization can lead to significant selection and sampling biases. The lack of allocation concealment, matched control groups, and blinding heightened the risks of allocation, recall, and reporting biases. Data integrity is also a concern, as quality control and assurance measures in real-world studies are typically less stringent compared to those in clinical trials [49-51]. Clinical trials are characterized by strong internal validity due to their rigorous design and clear protocols, but often with suboptimal external validity, limiting their generalizability. In contrast, real-world studies offer higher external validity and greater applicability by including a more diverse patient population reflective of everyday clinical settings [12-18].

Based on the available evidence, pembrolizumab monotherapy demonstrated a better overall response rate, and lesser adverse events, and similar PFS and overall survival compared to pembrolizumab combination therapy, while the latter did not significantly enhance the prognosis of patients. However, these findings are not conclusive due to the limitations of the studies included in the review. Additional robustly designed real-world research or prospective observational studies are needed to validate the role of pembrolizumab monotherapy vs. combination therapy in routine clinical practice.

## Conclusions

Our analysis showed improvement in overall response rate and lower incidence of adverse events with pembrolizumab monotherapy compared to pembrolizumab plus chemotherapy. However, no conclusion can be drawn on PFS and overall survival. Real-world studies provided a direct comparison of pembrolizumab monotherapy with pembrolizumab combination therapy. To our understanding, this is the first study to systematically review and aggregate data for a meta-analysis to evaluate the quality of evidence on the effectiveness and safety of pembrolizumab in real-world scenarios.

Although we conducted a comprehensive search to include all studies that met our eligibility criteria for this review, we may have missed one or two studies. Based on the available evidence, this review showed some beneficial effects of pembrolizumab monotherapy in HNSCC patients in routine clinical practice. However, due to the inherent biases of the studies included in the review, its utility as a standalone treatment requires more conclusive evidence. Further real-world studies or prospective observational studies should be conducted to substantiate the role of pembrolizumab in routine clinical settings.

## Appendices

Appendix A

**Table 5:** Data collection form.

Data Collection Form
Author –
Year -
Place -
Year of publication –
Duration of the study –
Design of the study –
Inclusion criteria -
Exclusion criteria -
Sample size -
Number of participants in Intervention arm –
Number of participants in Control arm –
Patient Characteristics –
Risk of bias assessment:
Selection of participants –
Confounding variables –
Intervention measurement –
Blinding of outcome assessment –
Incomplete outcome data –
Selective outcome reporting -
Outcome –
Overall response rate –
Progression-free survival-
Overall survival –
Adverse event/reaction -
Follow-up
Additional information –
P – Pembrolizumab
CT - Control
ORR – Overall Response Rate
PFS – progression-free survival
OS – Overall survival
Adverse event -
Cox Proportional Hazard Ratio –

Study	Number of participants		ORR		OS		ADR		PFS		Follow up (Months)	
Author, year	P	P+CT	P	P+CT	P	P+CT	P	P+CT	P	P+CT	P	P+CT
Black et al. 2023												
Cirillo et al 2024												
Hobday et al. 2022												
Iwaki et al. 2024												
Matsuo et al. 2023												
Nakano et al. 2022												
Okada et al. 2023												
Sano et al. 2022												

Appendix B

**Table 6:** Detailed search results from four databases PubMed, Embase, Scopus, Web of Science, and Cochrane Central. PICO: P – Metastatic/unresectable/recurrent head and neck cancer patients I – Pembrolizumab with or without chemotherapy (FU and Platinum-based therapy) C – Standard of care (FU and Platinum-based therapy) with or without placebo O – Survival

Keyword/ combination	PubMed	Scopus	Cochrane	Embase	Web of Science
(((((("Head and neck cancer " OR "Head and neck squamous cell cancer" OR " HNSCC" OR "recurrent head and neck cancer" OR "unresectable head and neck cancer" OR "metastatic head and neck cancer")) OR ("Head and Neck Neoplasms"[Mesh])) OR ("head neck neoplasm")) OR ("neck neoplasm")) OR ("head neoplasm")) AND (((Pembrolizumab OR "programmed death receptor-1 blocking antibody" OR "PDL1 blocking antibody" OR "programmed death receptor-1 inhibitor" OR "PDL1 inhibitor" OR "immune check point inhibitor")) OR ("pembrolizumab" [Supplementary Concept]))	739	1748	265	2341	791
Total					5884
